# Crosstalk between *Mycobacterium tuberculosis* and SARS-CoV-2 defines divergent cellular composition of murine responses

**DOI:** 10.3389/fimmu.2026.1895152

**Published:** 2026-09-03

**Authors:** Rachel E. Hildebrand, Madeleine B. Lodes, Shaswath S. Chandrasekar, Chungyi H. Hansen, Elizabeth Elsmo, Anthony J. Veltri, Adel M. Talaat

**Affiliations:** 1 Department of Pathobiological Sciences, School of Veterinary Medicine, University of Wisconsin, Madison, WI, United States; 2 Wisconsin Veterinary Diagnostic Laboratory, Madison, WI, United States; 3 Bioinformatics Resource Center, University of Wisconsin Biotechnology Center, Madison, WI, United States; 4 Vireo Vaccines International, Middleton, WI, United States

**Keywords:** co-infection, immunopathology, murine model, *Mycobacterium tuberculosis*, SARS-CoV-2, spatial transcriptomics

## Abstract

**Introduction:**

*Mycobacterium tuberculosis* infects nearly a quarter of the world population, while SARS-CoV-2 has infected hundreds of millions in recent years. The cellular and genetic impact of this coinfection remains unclear.

**Methods:**

Using a murine model, we studied SARS-CoV-2 superinfection during acute and chronic stages of high dose tuberculosis (TB).

**Results:**

In acute TB, coinfection increased mortality associated with higher brain viral loads. During chronic TB, survival improved slightly without affecting brain infection. Interestingly, histopathology showed disorganized inflammatory responses in co-infected mice versus granuloma-like structures in TB alone. Spatial transcriptomics revealed expanded B cell populations and upregulation of immunoglobulin-associated genes, as well as an absence of distinct Tcell clusters, consistent with a relative prominence of humoral immune signatures in chronic TB. Notably, SARS-CoV-2 infection produced bronchial inflammation dominated by innate immune cells, like acute TB but unlike the infiltration of foamy macrophage during chronic TB.

**Discussion:**

Overall, SARS-CoV-2 coinfection was associated with a distinct pulmonary immune landscape compared to TB alone, including differences in macrophage and lymphocyte composition and spatial organization, suggesting that coinfection may modulate the local immune response to TB and highlighting the need for further investigation into the development of vaccines and therapies targeting both pathogens.

## 1 Introduction

In March 2020, the World Health Organization (WHO) declared coronavirus disease 2019 (COVID-19), caused by severe acute respiratory syndrome coronavirus 2 (SARS-CoV-2), a global pandemic (1, 2). However, COVID-19 is not the only ongoing pandemic. Tuberculosis (TB), caused by *Mycobacterium tuberculosis* (*M. tuberculosis*), remains a leading cause of death from an infectious agent, with approximately 1.5 million deaths annually and an estimated one quarter of the global population harboring infection (3). The convergence of these two pandemics has raised significant public health concerns, as coinfection is increasingly recognized as a contributor to adverse clinical outcomes. Individuals with latent or acute TB exhibit higher mortality rates and prolonged recovery following SARS-CoV-2 infection compared with single-pathogen infections (4, 5). As expected, the COVID-19 pandemic disrupted TB diagnosis and treatment worldwide. In its first year, reported TB cases fell by 18%, primarily due to reduced healthcare access. In 2021, global TB mortality increased by 7.5%—the first rise since 2005 (3, 6). By 2023, TB incidence reached 8.2 million new cases, the highest since WHO began systematic reporting in 1995, surpassing COVID-19 as the leading infectious cause of death (7). Despite the public health emergency over COVID-19 being lifted by the WHO, millions of new cases and thousands of deaths are being recorded by the WHO dashboard, caused by variants of SARS-CoV-2. Although the WHO lifted COVID-19’s Public Health Emergency of International Concern, SARS-CoV-2 continues to circulate globally, with millions of new cases and thousands of deaths each year driven by emerging variants. Investigating the interplay between SARS-CoV-2 and *M. tuberculosis* infections is challenging due to the complexity of accurately modeling human TB pathogenesis in animals. In humans, only ~10% of infections progress to acute disease, while the remaining ~90% remain latent (latent TB infection, LTBI) (8–11). LTBI serves as a reservoir for reactivation when immune competence declines (9, 10). *M. tuberculosis* and SARS-CoV-2 both modulate host immunity, albeit via distinct mechanisms. LTBI and non-progressive TB are characterized by a balance between pro- and anti-inflammatory responses, with key cytokines including IFN-γ, TNF-α, IL-10, and IL-17 (12). Granuloma formation exemplifies this balance, containing bacilli while limiting immunopathology (13). In contrast, SARS-CoV-2 infection often induces hyperinflammation and lymphopenia, resulting in severe immune dysregulation (14). The potential for these divergent immune responses to synergize or antagonize during coinfection remains poorly understood. To address this gap, microbial and histological analyses followed by spatial transcriptomics (ST) were employed to characterize the pulmonary microenvironment in *M. tuberculosis*–SARS-CoV-2 coinfection on genetic and cellular levels.

Despite the importance and potential impact of understanding the tuberculosis/SARS-CoV-2 superinfection, only a few studies exploring the coinfection in animals have been published (15–17). Earlier reports suggested that *M. tuberculosis*-induced lung changes may inhibit early SARS-CoV-2 replication and subtly alter cytokine profiles. However, infection with more virulent *M. tuberculosis* strains has been associated with increased bacterial burdens and exacerbated lung pathology in co-infected hosts (17). As suggested earlier, the extent and nature of host immune modulation were dependent on the mouse/virus model utilized as well as whether the mice were chronically or recently infected with virulent strains of *M. tuberculosis* (15). Here we used the pathogenic *M. tuberculosis* Erdman strain to investigate the impact of SARS-CoV-2 superinfection during distinct TB disease stages. Mice infected with a high *M. tuberculosis* inoculum were subsequently superinfected with SARS-CoV-2 during the acute phase (4 weeks post-infection, WPI) or chronic phase (16 WPI) of TB. We observed that SARS-CoV-2 superinfection during acute TB resulted in 100% mortality and significantly elevated brain viral loads. In chronic TB, coinfection induced extensive, disorganized pulmonary inflammatory responses compared to TB-only controls. Host spatial transcriptome analysis revealed distinct immune and cellular responses determined by both TB disease stage and SARS-CoV-2 coinfection status, highlighting complex bacterial–viral–host interactions in the lung microenvironment.

## 2 Materials and methods

### 2.1 Cells and viruses

The Vero E6 cells were maintained at 37 °C, 125 rpm, and 5% CO_2_ in Dulbecco’s modified Eagle’s medium (DMEM) with 10% fetal bovine serum (FBS) and penicillin–streptomycin. Vero E6 cells were used to propagate SARS-CoV-2 isolate USA_WA1/2020 (lineage A) which was then titrated using the TCID_50_ method and expressed as plaque-forming units (PFU). The WA1/2020 strain was selected as the reference strain for SARS-CoV2 infection in the K18-hACE2 model to allow a direct comparison with earlier reports on this model strain from our group (18) and others (19).

### 2.2 Bacterial strains and growth conditions

*M. tuberculosis* Erdman was grown in Middlebrook 7H9 medium (Remel, Lenexa, KS) with 10% albumin–dextrose–catalase (ADC) and 0.05% Tween 80 to an optical density (OD_600_) of ~1.0. After two washes with PBS, the cultures were stored in storage medium (10% glycerol, 0.05% Tween 80, and 0.85% NaCl in deionized water) and stored at -80 °C. The infection dose was determined via plating. For infection, cultures were resuspended in PBS. Mycobacterial isolation from tissues was determined via homogenization in PBS and then plating on Middlebrook 7H10 medium (Remel, Lenexa, KS, USA) supplemented with 10% ADC and 10 mg/L amphotericin B, an antifungal agent (17). The plates were incubated for 4–6 weeks, with negative plates left for 8 weeks to confirm absence of growth.

### 2.3 Mouse infections

The effects of *M. tuberculosis* superinfection with SARS-CoV-2 were evaluated in transgenic C57BL/6 K18 hACE2 mice (6 to 8 weeks old) obtained from Jackson Laboratory and maintained in biosafety level 2 or 3 as appropriate. The mice were infected with *M. tuberculosis* via aerosol using the Glas-Col (Glas-Col, LLC, Terre Haute, IN, USA) inhalation exposure system as previously described (20). The target dose for the infection was 10^3^ CFU/lung. A single mouse from each experiment was sacrificed the day after infection, and lungs were homogenized and plated to confirm dose. The mice were challenged with 10^3^ PFU SARS-CoV-2 (50% lethality in this model) intranasally at 4 and 16 weeks post-infection with *M. tuberculosis.* The mice were weighed on the day of SARS-CoV-2 infection and every day thereafter. At 4 days after SARS-CoV-2 challenge or when considered moribund as determined by severe lethargy or at a greater than 20% reduction in weight, the mice were euthanized humanely via isoflurane overdose and cervical dislocation. Uninfected control mice were also sacrificed in conjunction with the superinfected mice. The lung, spleen, liver, kidney, and brain were collected for spatial RNA sequencing, histopathology, colony counts, and viral load assays. Two mice died of *M. tuberculosis* alone prior to the 16 WPI time point.

### 2.4 Viral load enumeration

Tissue samples were weighed and then homogenized via bead-beating to a total volume of 1 mL in serum-free medium (Opti-MEM). Bead-beating was performed with sterile zirconia beads, and the samples were clarified by centrifugation at 800 x *g* for 10 min at 4 °C. Viral titers were determined via growth in Vero E6 cell monolayers. The Vero E6 cells were seeded at 25,000 cells/well and then incubated overnight at 37 °C. A serially diluted tissue suspension was added in technical quadruplicates for 1 h at 37 °C, and then the medium was replaced with fresh DMEM. Plates were incubated in the same conditions for 3 days, after which the cytopathic effect (CPE) was observed microscopically at ×40 magnification. Viral titers were then expressed using TCID_50_ per gram tissue converted to PFU as done previously (18).

### 2.5 Histopathology and spatial transcriptome profiling

Samples of the lung, spleen, and liver were sectioned and stained with hematoxylin and eosin (H&E). They were then evaluated by a trained pathologist blinded to sample identity. Formalin-fixed paraffin embedded (FFPE) samples were sent for spatial sequencing using 10x Genomics’ Visium CytAssist (10x Genomics, Pleasanton, CA, USA). Briefly, thin sections (3–10 µm) of FFPE tissue were cut and imaged. Tissue sections were hybridized with barcodes to account for spatial location as well as probes for the murine transcriptome *mm10-2020-A* on an 11-mm Visium V5 slide (21). Hybridized mRNA was allowed to extend and was washed off. Libraries were then constructed and sequenced with an Illumina NovaSeq X Plus sequencer (Illumina, San Diego, CA, USA) to an average of 66,796 reads per barcoded location on the tissue (spot). An average of 19,346 genes was detected under each tissue, and the average median genes per spot count was 4,972. After sequencing, samples were processed using the Space Ranger count pipeline to align barcodes to high-resolution hematoxylin and eosin (H&E) images and to correct for sequencing depth and other quality control metrics. Space Ranger Aggregate was used to aggregate all samples into a single Loupe file for differential expression analysis.

Differential expression analysis was first performed between treatment groups and PBS controls using Loupe Browser 8.0.0 (https://www.10xgenomics.com/). Resulting differentially expressed gene sets were then filtered to include only genes with a log2 fold change of greater than 2.0 or less than -2.0 and a *p*-value of less than 0.001. Fold change values were calculated using the aggregated Loupe file to select each sample as its own cluster, and then we used the differential expression tool (exact negative binomial test) to compare between treatment samples and PBS controls. All gene set *p*-values were adjusted using Benjamini-Hochberg correction for multiple tests.

Samples were loaded into Seurat (22) using *Load10X_Spatial* and filtered to remove spots covering non-lung tissue. Samples were merged by time point using Seurat’s merge function and normalized using SCTransform and batch corrected using Harmony. UMAP plots were then created using Seurat’s RunUMAP function. Cell type populations were annotated using the ScType mouse marker database filtered for immune and lung cell types. Module scoring as well as Loupe Browser deconvolution genes were used to confirm annotation of cell types for each cluster. Differential composition analysis was performed using sccomp’s Bayesian hierarchical model to compare groups with variable sample sizes to PBS control(s) (23). Moran’s I was used to measure spatial autocorrelation of cell types separately for each tissue sample. A custom R function was used to calculate Moran’s I, with significance assessed using z-scores and Benjamini-Hochberg FDR correction for multiple testing. A minimum of 10 spots per cell type per sample was needed for calculation. Macrophage populations were investigated using a gene set for both classical (Adgre1, Cd68, Itgam, Mrc1, Cd14 and Csf1r) and foamy (Apoe, Plin2, Plin3, Lpl, Fabp4, Trem2 and Lipa) macrophage groups. A box plot was created to show the distribution of raw module scores for classical and foamy macrophage gene sets across all spots, grouped by infection and time point. T cell subset module scores were determined in each cluster using Seurat’s AddModuleScore. CD4 T cell signature was detected with a gene set containing Cd4, Trac, Trbc1, Il2ra, Foxp3, Cd3e, Cd3d, Tcf7 and Lef1. CD8 T cell signature was detected with a gene set containing Cd8a, Cd8b1, Gzmb, Prf1, Ifng, Gzma, Nkg7, and Klrg1.

Pathway enrichment analysis was done using the R package clusterProfiler’s *gseGO* (24). The genome wide annotation for mouse, org.Mm.eg.db, was used to annotate enriched pathways of biological process (BP). Differential expression analysis was similarly performed between treatment groups and PBS controls using Loupe Browser 8.0.0 before analysis with *gseGO*. Dot plots were made using the enrichplot package. The Benjamini-Hochberg (BH) method was used during *gseGO* analysis to control for false discovery rate (FDR) and a p-value of 0.001 was selected as significant.

### 2.6 Statistical analysis

Statistical analysis was done using GraphPad Prism (GraphPad, La Jolla, CA) and Loupe Browser Benjamini-Hochberg adjusted p-values. Statistical analysis for bacterial and viral load used One-way ANOVA. UMAP clustering was done using the Seurat Louvain algorithm. P-values for **Figure 5** were taken directly from Loupe Browser differential expression (exact negative binomial test with Benjamini-Hochberg adjusted p-values) comparing treatment groups to each other. Cell type annotations were made using scType (deterministic ranking score based on curated cell type marker database). P-values for **Figure 7** were calculated using the R package sccomp (Bayesian Dirichlet-multinomial model) (23) with a significance threshold of FDR <0.05. Moran’s I was calculated using spatial autocorrelation to produce z-score, p-value and FDR. P-values <0.05 (*), <0.01 (**), <0.001 (***), and <0.0001 (****) were considered significant.

## 3 Results

### 3.1 Acute tuberculosis increases SARS-CoV-2-induced mortality

**Figure 1:**
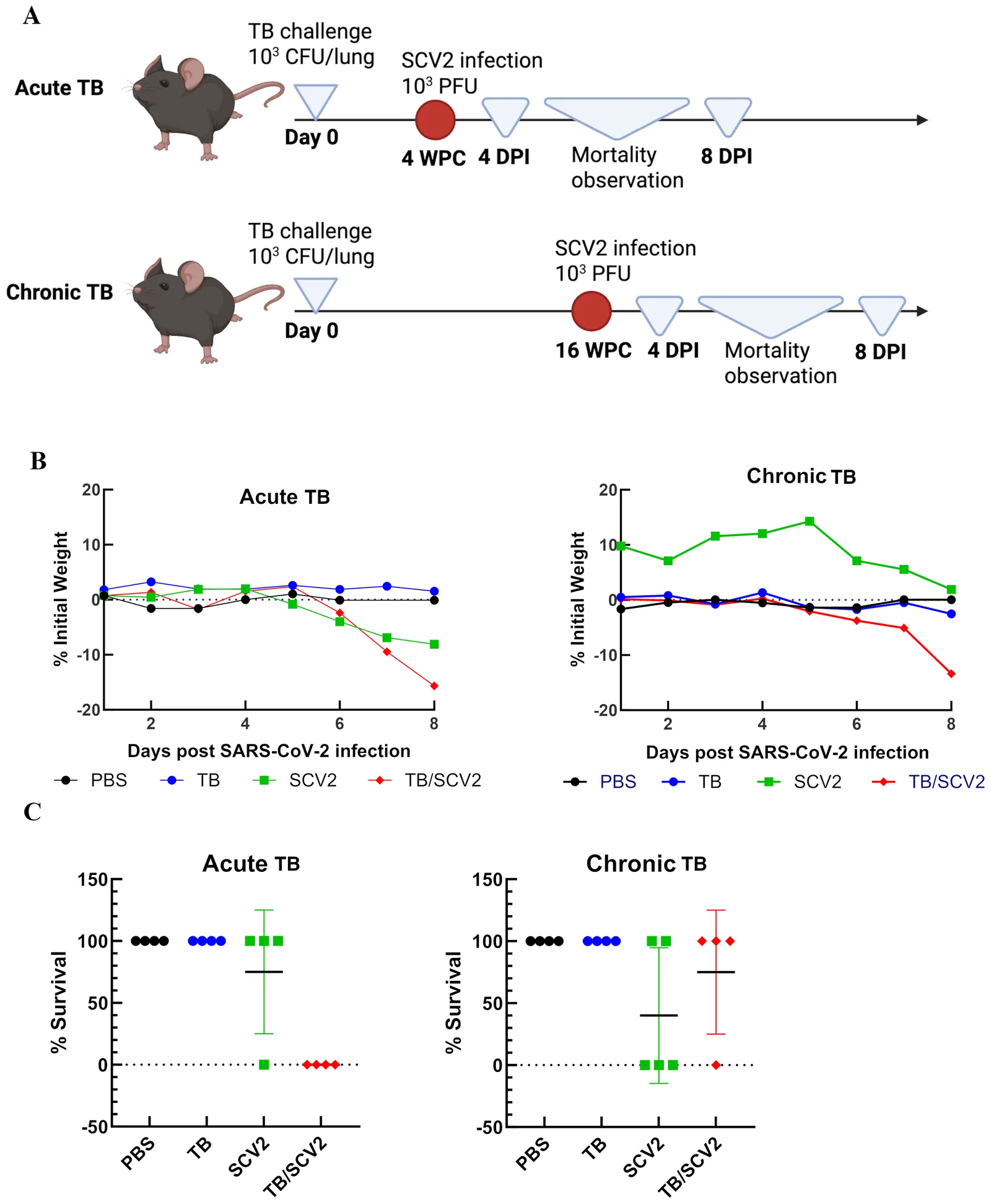
Differential survival of SARS-CoV-2 superinfected mice (K18-hACE2) depending on whether tuberculosis is at acute or chronic phases of infection. **(A)** Figure depicting study design, made with BiorenderTM. **(B)** Weight loss and gain as a percentage of initial weight (measured in g) at 4 WPI (acute) and 16 WPI (chronic) timepoints and **(C)** Percent survival of mice during mortality observation at 4 WPI (acute) and 16 WPI (chronic) with tuberculosis. Four to 6 mice per treatment group were analyzed. WPI = weeks post infection.

To assess the impact of SARS-CoV-2 superinfection on *M. tuberculosis* pathogenesis, K18-hACE2 mice were first infected with a high dose of aerosolized *M. tuberculosis* (10³ CFU/lung) to establish acute and chronic disease. At 4 and 16 weeks post-infection (WPI) representing both acute and chronic tuberculosis phases (25, 26), separate groups of *M. tuberculosis*-infected mice were intranasally inoculated with a high dose of SARS-CoV-2 (10³ PFU). The animals were weighed daily and monitored for 8 days post-SARS-CoV-2 infection (DPI) (**Figure 1A**). During acute tuberculosis (4 WPI), the co-infected mice started to lose weight steadily from 5 to 6 DPI that reached down to ~15% of the co-infected animals compared to only 10% reduction in body weight of SARS-CoV-2 mice. Strikingly, mortality reached 100% in the *M. tuberculosis*–SARS-CoV-2 co-infected group, compared with only 25% mortality in SARS-CoV-2-only controls (**Figures 1B, C**). In contrast, during the chronic phase (16 WPI), weight loss in co-infected mice began earlier (4 DPI) but progressed more gradually, reaching ~15% similar to the acute phase. Unlike acute tuberculosis, ~60% of co-infected mice at chronic tuberculosis survived to the study endpoint, compared with ~40% survival in SARS-CoV-2-only mice. As expected, PBS-treated controls did not exhibit morbidity or mortality at either disease stage. Although two tuberculosis-only-infected mice died prior to the 16-week post infection time point, the majority of *M. tuberculosis*-only mice did not exhibit morbidity or mortality at either disease stage, suggesting that coinfections could lead to deteriorating health conditions of the host at different rates depending on the tuberculosis phase of infection.

### 3.2 Prior *M. tuberculosis* infection results in reduced viral loads in murine lungs

**Figure 2:**
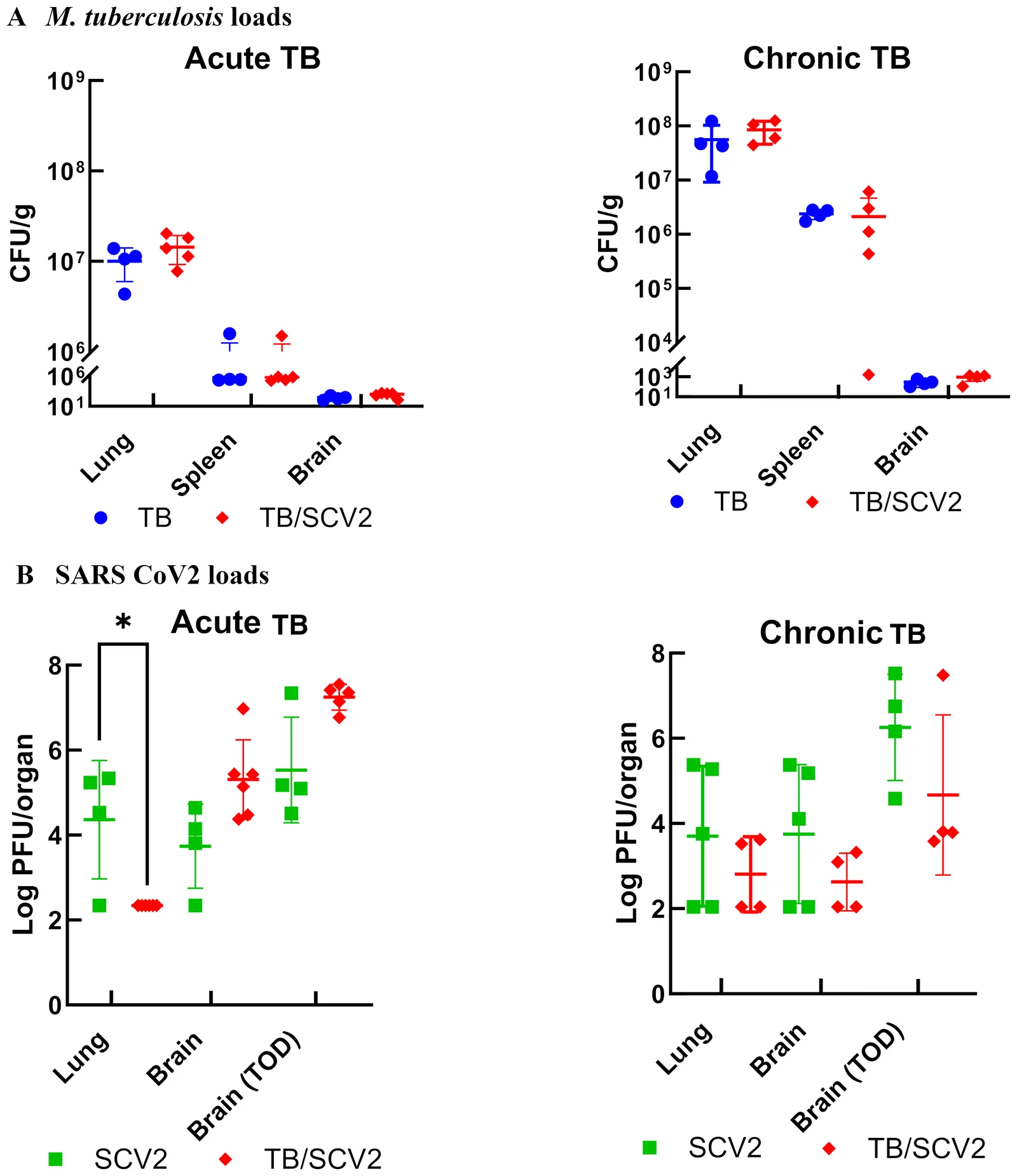
Pathogen loads in superinfected mice. **(A)** *M. tuberculosis* loads in murine lung, spleen and brain tissue presented as colony forming unit per gram from mice sacrificed during mortalityobservation between 5 and 8 DPI following infection with SARS-CoV-2 at both acute and chronic tuberculosis. **(B)** Viral loads in murine lungs, brains and brains at the time of death (TOD) (PFU per organ) from mice sacrificed at 4 DPI SARS-CoV-2 infection at the acute and chronic tuberculosis. Statistical analysis was performed in GraphPad Prism using One-way ANOVA. P-values were considered significant if p<0.05 (*). Four to 6 mice per treatment group were analyzed. ANOVA = analysis of variance; DPI = days post infection; PFU = plaque forming units

To determine whether prior *M. tuberculosis* infection influences SARS-CoV-2 replication, both bacterial and viral loads were quantified in the lungs, spleens, and brains of K18-hACE2 mice (*N* = 4–6/group) at 4 days post-SARS-CoV-2 infection (DPI)—the time point at which peak viral titers are typically observed in this model (27). As expected, bacterial loads were high in both lungs and spleen due to the initial 10^3^ dose of *M. tuberculosis* Erdman (**Figure 2A**). No significant differences in bacterial burden were observed between the tuberculosis-only and tuberculosis/SARS-CoV-2 coinfection groups in both acute and chronic tuberculosis. However, consistent with previous reports (14, 17), prior *M. tuberculosis* infection significantly reduced lung SARS-CoV-2 levels during acute tuberculosis (**Figure 2B**), suggesting a degree of protection against concurrent SARS-CoV-2 replication where the SARS-CoV-2 levels were at or near the detection limit for all co-infected murine lungs. In brains, prior *M. tuberculosis* infection during the acute phase was associated with higher brain PFU levels compared with SARS-CoV-2-only controls at the time of sacrifice and time of death (TOD), although this did not reach statistical significance. Conversely, during the chronic phase, *M. tuberculosis*-primed mice exhibited lower PFU levels in lungs and brains than SARS-CoV-2-only mice, but this did not reach statistical significance.

### 3.3 Reduced number of organized foci and inflammation with SARS-CoV-2 superinfection

**Figure 3:**
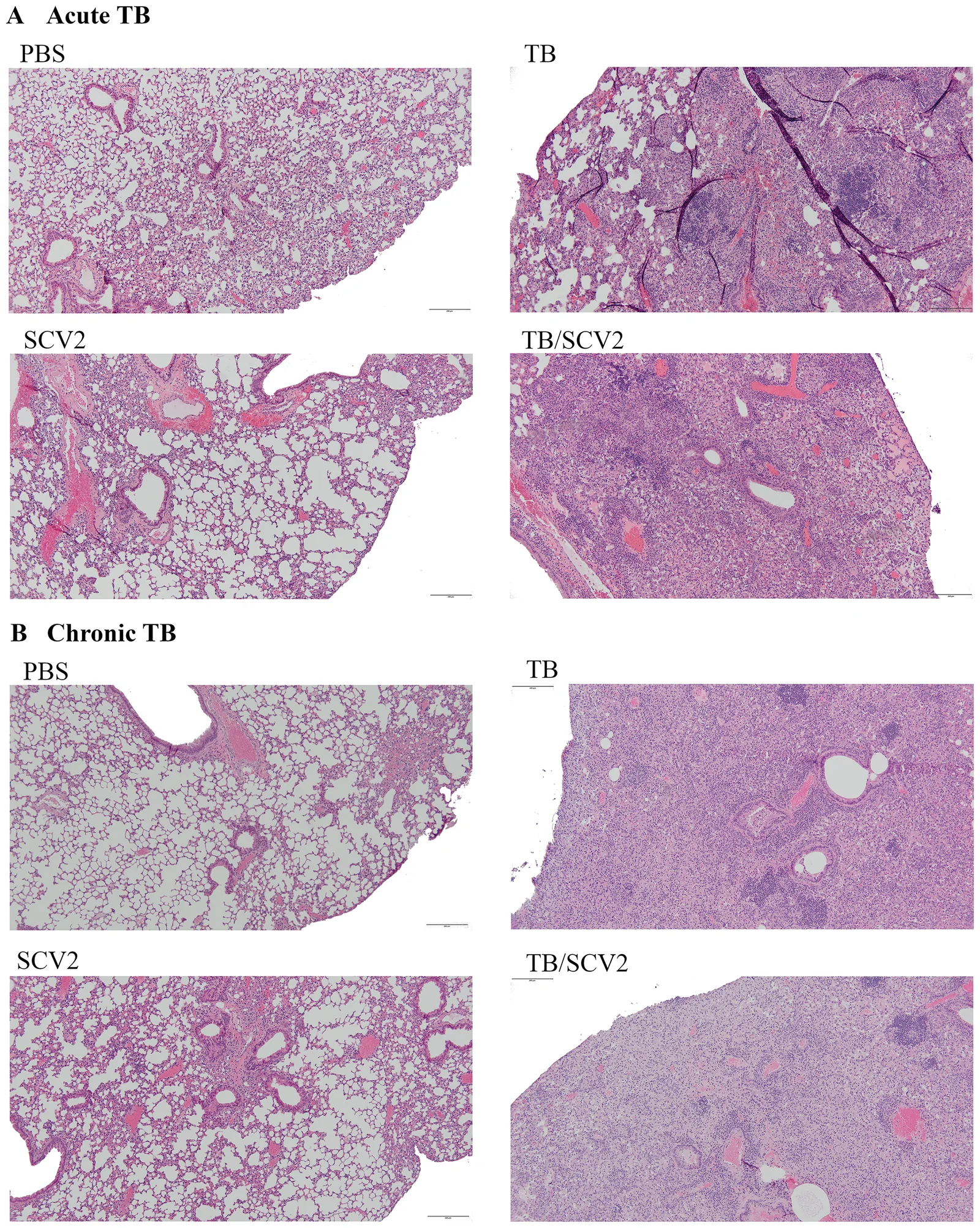
Reduced number of organized foci and inflammation with SARS-CoV-2 coinfection. Murine lung tissue sections stained with hematoxylin and eosin (H&E) are shown above from **(A)** Mice sacrificed at acute infection with *M. tuberculosis* and **(B)** Mice sacrificed at chronic infection with *M. tuberculosis*. All mice were sacrificed in-between days 5-8 after SARS-CoV-2 or PBS (mock) infection at their respective *M. tuberculosis* timepoint. Four to 6 mice per treatment group were analyzed. A representative image was selected for each treatment from each time point of infection. PBS = phosphate buffered saline.

Lungs were collected from both moribund and surviving mice throughout the mortality observation period to assess histopathological changes associated with SARS-CoV-2 superinfection. Animals were selected for lung, spleen, and liver analysis by a trained pathologist from each treatment group on a blinded basis. The lung lesions observed in tuberculosis-only and SARS-CoV-2 groups were consistent with each infection, respectively, where granuloma-like structures were observed at both acute and chronic tuberculosis, while only mild to notable lymphocytic infiltrations were seen in SARS-CoV-2 lungs (**Figure 3A**) as determined by the trained pathologist. Interestingly, widespread lymphocytic and histiocytic infiltrates were observed in the tuberculosis/SARS-CoV-2 co-infected groups at both acute and chronic tuberculosis with fewer discrete, organizing granuloma-like inflammatory structures than were observed in their tuberculosis-only counter groups (**Figure 3B**), consistent with lesions observed earlier by our group and others (17, 21). Specifically, the co-infected lungs exhibited more heterogeneous populations of histiocytes admixed with lymphocytes (**Figure 3**). Organized granuloma-like structures were fewer and occupied less tissue area in the acute compared to chronic tuberculosis lungs, with acute stage lungs receiving severe to severe-spread scores for granulomatous formation and chronic stage lungs receiving mostly severe-spread scores by a trained pathologist. The liver and spleen sections showed a notable level of granulomatous responses in groups infected with *M. tuberculosis* at both acute and chronic phases of tuberculosis, overall increasing from mild to notable granulomatous response from acute to chronic infection stage. Co-infected liver and spleen sections at acute tuberculosis had variable severity levels of lymph infiltration and organized inflammation that became more consistently granulomatous in nature at the chronic time point of infection. Overall, differences between tuberculosis-only and tuberculosis/SARS-CoV-2 groups during both acute and chronic phases were subtle, primarily reflecting the more heterogenous populations of inflammatory cells with fewer well-organized aggregates of histiocytes in the co-infected groups. The tuberculosis-only groups exhibited larger and more organized histiocytic foci than were observed in the co-infected groups.

### 3.4 Spatial RNA sequencing analysis for host–pathogen interactions during superinfection

To elucidate the cellular landscape responding to superinfection in murine lungs, we performed spatial RNA sequencing on lung sections from mice at acute and chronic tuberculosis stages using the 10x Genomics Visium CytAssist platform. Gene expression profiles of over 19,000 murine genes (19,410 for acute stage and 19,397 for chronic stage, mouse GenBank dataset) were analyzed across 16 tissue sections from acute and chronic coinfection experiments. Two lung samples from each treatment group were selected for spatial sequencing apart from the acute stage of TB infection in which three samples were submitted from the co-infected group and one from the PBS control group. Data were normalized for sequencing depth, and batch effects were corrected via the Space Ranger pipeline. Quality control metrics indicated high sequencing quality across samples, and violin plots of gene and UMI counts per spot are shown in **Supplementary Figure 1**.

**Figure 4:**
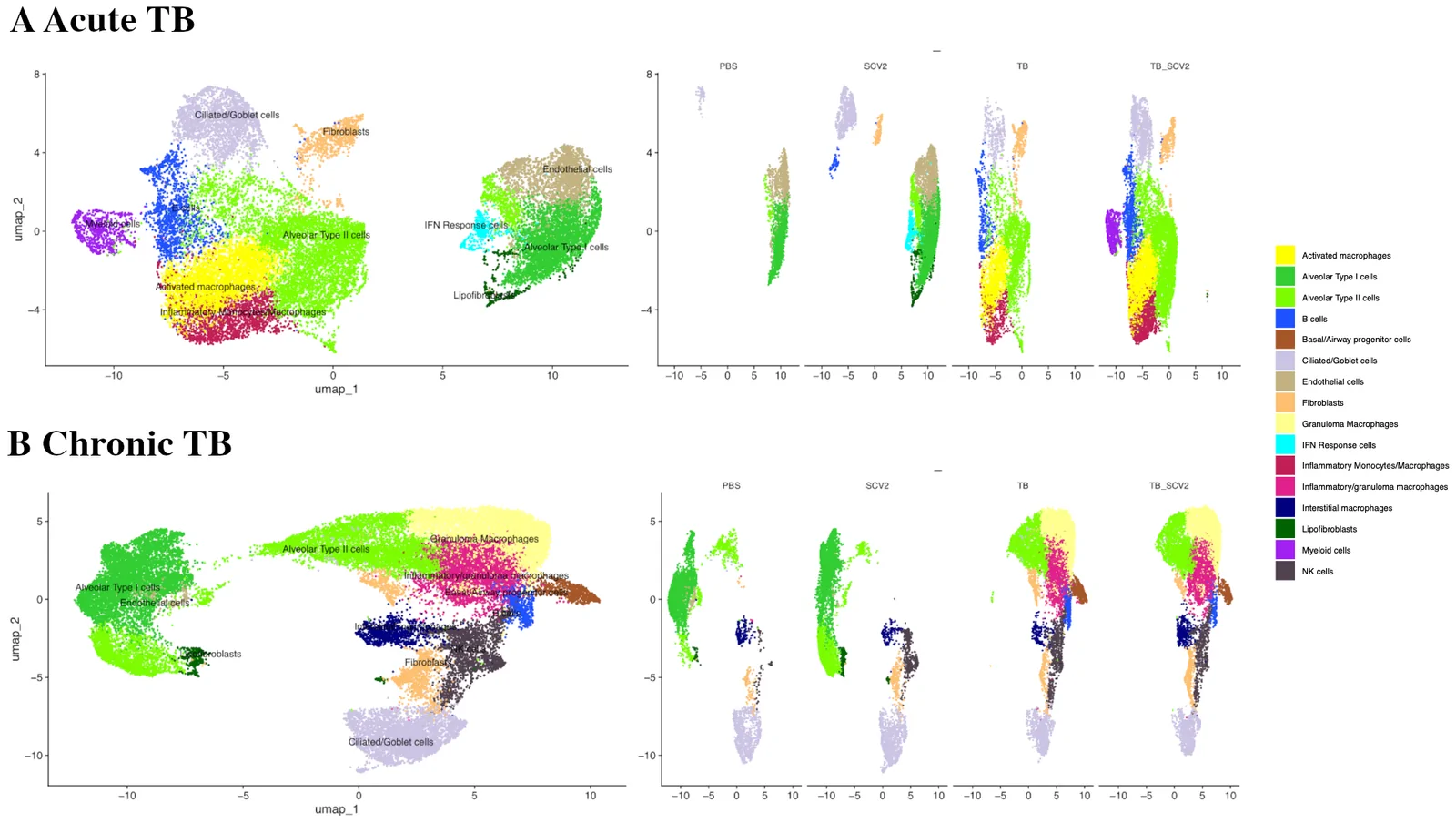
UMAP gene expression analysis performed by Visium CytAssist of murine lungs. UMAP clustering was run on both acute and chronic time points of infection using Seurat Louvain algorithm. Clustering is shown both combined and split by treatment for **(A)** acute infection or **(B)** chronic infection with *M. tuberculosis*. Lung samples (N=2) per treatment group were analyzed, aside from the acute stage of tuberculosis infection in which 3 samples were analyzed for the co-infected group and 1 for the PBS control group. UMAP = Uniform Manifold Approximation and Projection.

To understand the trends of gene expression and cell type clustering between treatments at acute and chronic stages of tuberculosis infection, UMAP plots were generated via Seurat. UMAP analysis showed high similarity in cell type clusters for uninfected PBS samples and SARS-CoV-2-only-infected samples at both time points of infection, though at acute infection the SARS-CoV-2-only-infected samples showed some B cell, IFN response cells, and fibroblasts not seen in the PBS sample at that time point (**Figure 4**). A similar trend was seen in the tuberculosis-only and superinfected groups, where the superinfected group contained a myeloid cell cluster not seen in tuberculosis-only-infected tissue at acute infection, while both tuberculosis-infected groups approached similar cell type groupings at the chronic time point of infection. The SARS-CoV-2-only samples closely aligned with PBS controls at both time points, especially at chronic tuberculosis infection, suggesting a low level of transcriptome-wide divergence from the controls.

**Figure 5:**
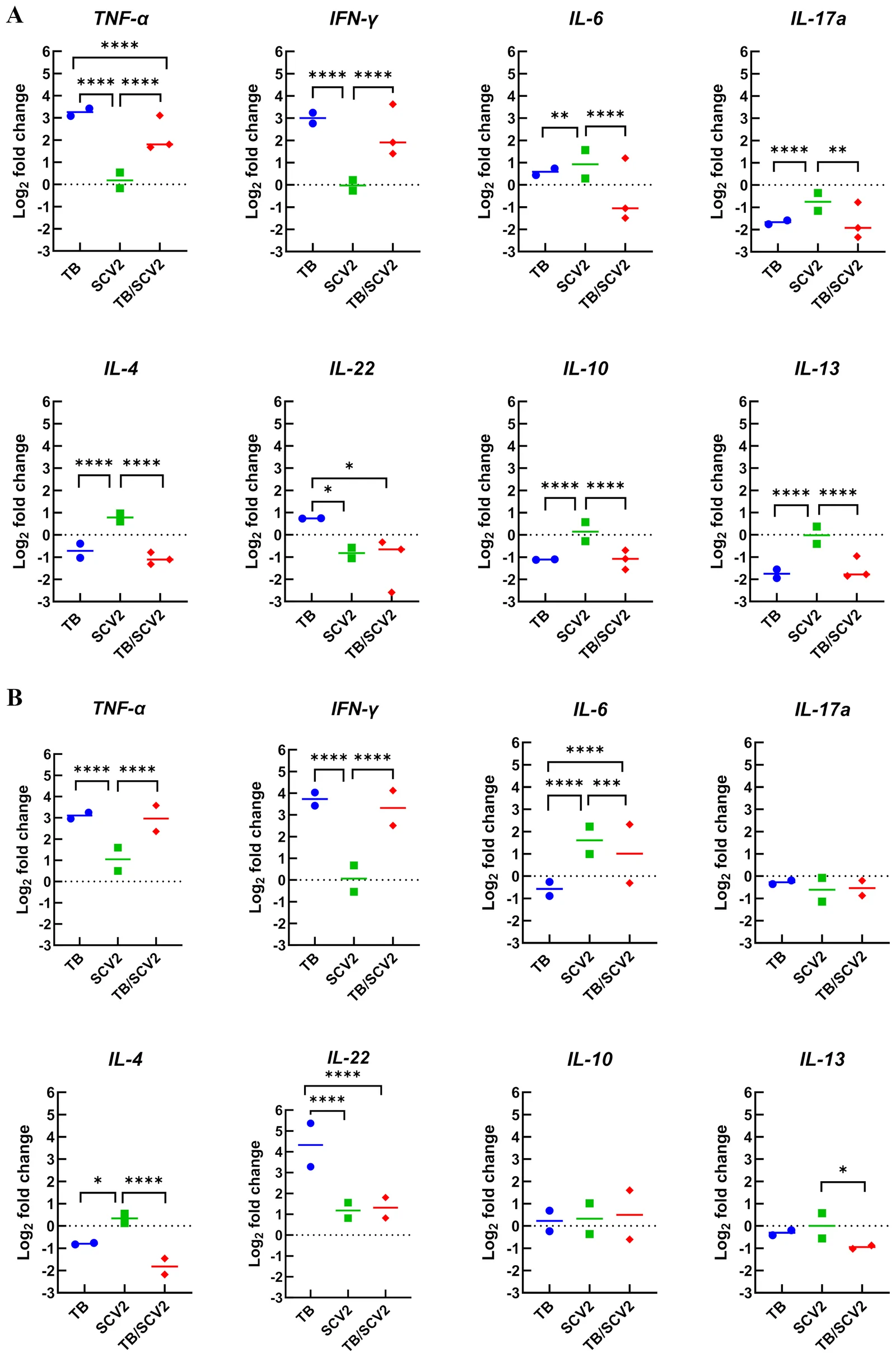
Differential expression of key adaptive immune-related genes in murine lungs. The expression of cytokines calculated as log2 fold change compared between samples and taken at **(A)** acute infection or **(B)** chronic infection with *M. tuberculosis*. Calculations were performed using Loupe Browser 8.0.0 using the differential expression calculation tool (exact negative binomial test) to examine the entire tissue. Samples were compared relative to a PBS control. Statistical analysis was performed in Loupe Browser using the negative binomial test to compare adjusted p-values between treatments. P-values were considered significant if p<0.05 (*), p<0.01 (**), p<0.001 (***), and p<0.0001 (****). Two lung samples per treatment group were analyzed, aside from the acute stage of tuberculosis infection in which 3 samples were analyzed for the co-infected group and 1 for the PBS control group. PBS = phosphate buffered saline.

**Figure 6:**
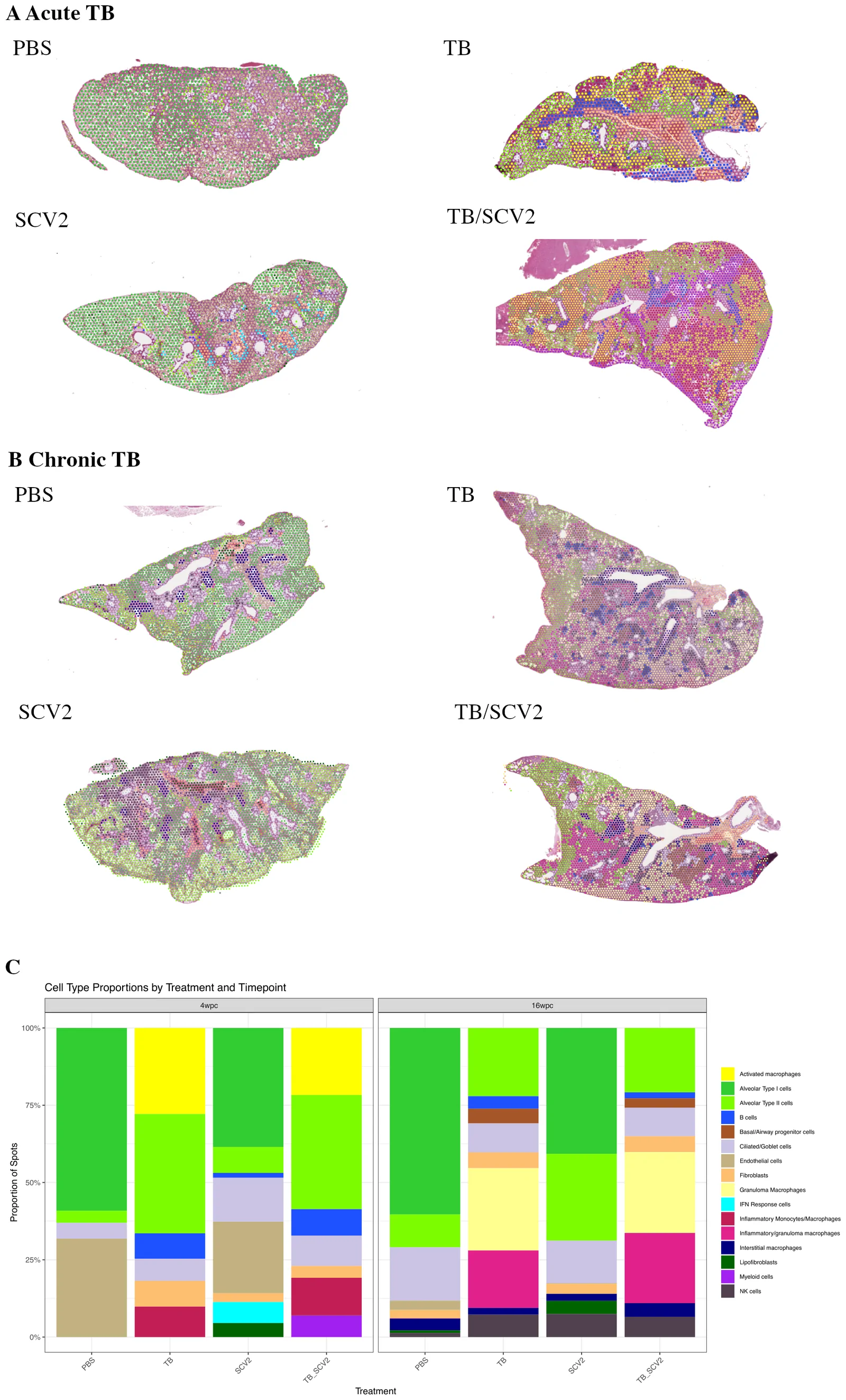
Dominant cell types represented in murine lungs. Cell type annotations done using ScType restricted to immune and lung cell types to determine dominant cell type for each cluster. Loupe Browser deconvolution clusters were used to confirm ScType annotations. Spatial distribution of cell types as well as proportions of spots for each cell type for **(A)** acute infection or **(B)** chronic infection with M. tuberculosis are shown. Cell type proportions are shown for both acute and chronic time points in **(C)**. Only one replicate is shown per treatment in panels **(A, B)** for clarity. Two lung samples per treatment group were analyzed, aside from the acute stage of tuberculosis infection in which 3 samples were analyzed for the co-infected group and 1 for the PBS control group. PBS = phosphate buffered saline.

**Figure 7:**
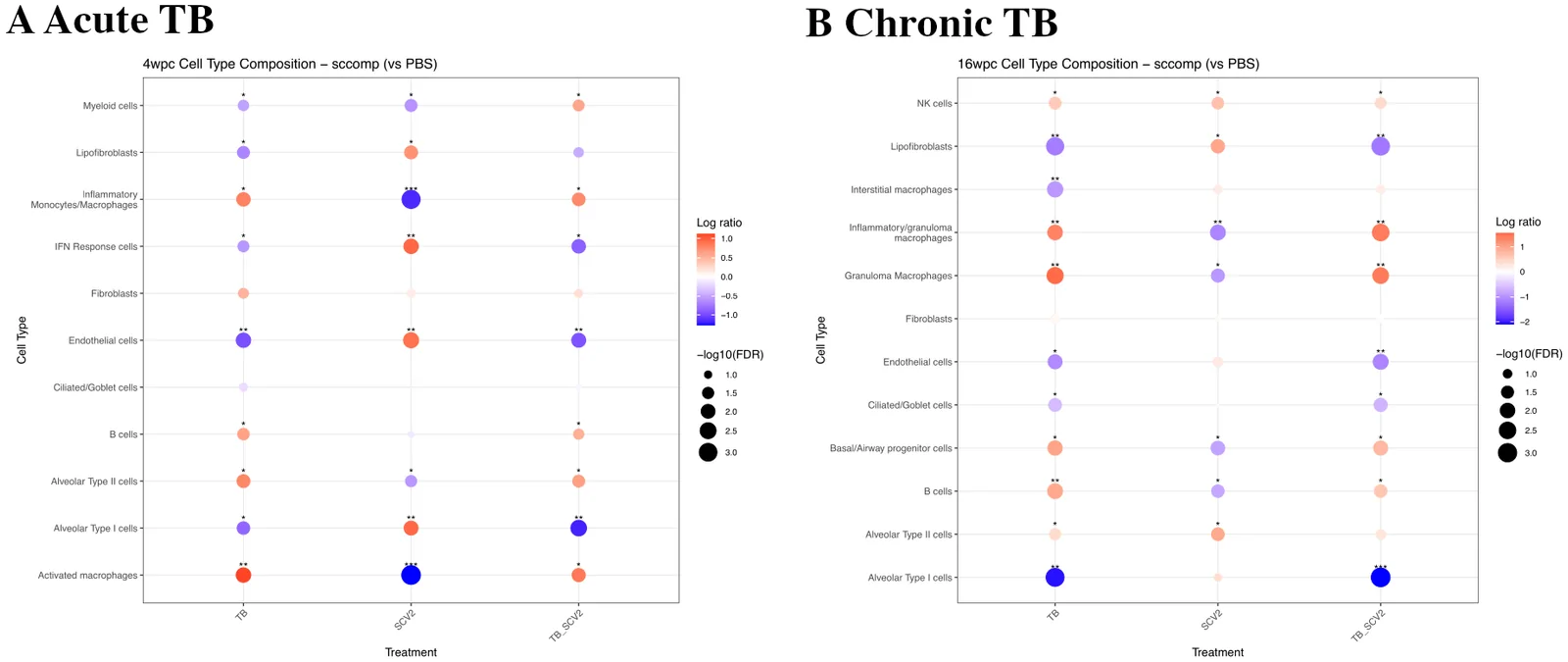
Spatial RNA-seq Analysis of cells involved in host responses to infections among murine groups. Differential composition analysis performed using sccomp (Bayesian Dirichlet-multinomial model) to compare enrichment or depletion of cell type groups compared to PBS control(s) at **(A)** acute infection or **(B)** chronic infection with *M. tuberculosis*. An FDR of < 0.05 was used. Results were considered significant if FDR<0.05 (*), FDR<0.01 (**), or FDR<0.001 (***). FDR = false discovery rate; PBS = phosphate buffered saline

### 3.5 Unique gene expression profile for murine-infected and super-infected groups

Following general UMAP analysis, more targeted gene expression analysis was done. Among the analyzed samples, gene expression levels for all spots under the tissue were compared among samples to levels expressed in PBS controls using Loupe Browser’s differential expression tool (exact negative binomial test) and filtered for log2 fold changes ≥|2.0| and *p* ≤ 0.001 (Benjamini–Hochberg adjusted). At acute tuberculosis, a total of seven genes overlapped all infection groups: Ccl8, Cxcl10, Reg3g, Muc5b, Tnnt2, Ifit1, and Bpifb1. Most of these genes are involved in innate immune response and were upregulated. Most overlapping genes (*n* = 251) occurred between tuberculosis-only and superinfected samples, with few overlaps occurring between superinfected or tuberculosis and SARS-CoV-2 samples (**Table 1**), suggesting a low-level impact of SARS-CoV-2 infection. At chronic tuberculosis, a total of six genes overlapped between all infection groups: Kap, Slc34a1, Acsm2, Miox, Slc22a8, and Akr1c21. All of these genes were downregulated and unrelated to immune response. Similar to acute tuberculosis, most of the gene overlaps (*n* = 374) existed between superinfected and tuberculosis-only-infected samples at the chronic stage of infection. No gene overlaps existed between SARS-CoV-2-only lungs and tuberculosis or co-infected lungs between time points, which is a further indication of the unique nature of host responses to each infection and coinfection at the chronic phase of tuberculosis. Generally, upregulated genes dominated all infection groups at the acute time point, while downregulated genes predominated at the chronic time point (**Table 1**). Upregulated genes in tuberculosis-only and superinfected lungs mainly involved immune responses; SARS-CoV-2-only lungs exhibited upregulated genes related to normal cellular functions, with a limited contribution to the tuberculosis superinfected group with SARS-CoV-2.

**Table 1:** Gene expression analysis using Visium CytAssist for RNA sequence profiling.

Overlaps	TB			SCV2			TB/SCV2		
	Total genes	Upregulated genes	Downregulated genes	Total genes	Upregulated genes	Downregulated genes	Total genes	Upregulated genes	Downregulated genes
Acute									
Overlapping with TB	902	301	601	11	11	0	258	144	114
Overlapping with SCV2	11	11	0	33	33	0	8	7	1
Overlapping with TB/SCV2	258	144	114	8	7	1	337	161	176
Chronic									
Overlapping with TB	554	221	333	7	0	7	380	147	233
Overlapping with SCV2	7	0	7	37	29	8	7	0	7
Overlapping with TB/SCV2	380	147	233	7	0	7	584	165	419

Overlapping genes among murine lungs of SARS-CoV-2 superinfection and between acute and chronic tuberculosis. The total number as well as number of upregulated and downregulated genes are shown for each group. Differential expression was performed for each treatment sample compared to the PBS control(s) using Loupe Browser’s differential expression tool (exact negative binomial test). Differential expression gene sets were filtered to include only genes with log2 fold change of >2.0 or <-2.0 and a *p*-value of <0.001. *P*-values were adjusted using Benjamini–Hochberg correction. Two lung samples per treatment group were analyzed aside from the acute stage of tuberculosis infection in which three samples were analyzed for the co-infected group and one for the PBS control group.

PBS, phosphate-buffered saline.

### 3.6 Differential expression of key immune genes

To further shed light on immune kinetics identified for coinfection groups, we profiled the expression of key chemokine- and cytokine-related genes associated with innate and adaptive immunity during tuberculosis and SARS-CoV-2 infection using Loupe Browser v8.0.0 on the Visium data set. At acute tuberculosis, induced genes included chemo-attractants and chemokine receptor binders such as Saa3 (LFC ~6.9), Cxcl9 (~6.5), Ly6i (~6.5), Cxcl10 (~6.1), and Ccl8 (~6.7), all highly significant (*p* < 1.0E-50) (UniProt Knowledgebase database, UniProtKB). Among the genes overlapping between all treatment groups at acute infection, all were upregulated and related to immune response: Ccl8, Cxcl10, Tnnt2, Reg3g, Ifit1, Muc5b, and Bpifb1. Ccl8 and Bpifb1 were also found in both tuberculosis-only and co-infected lungs at chronic infection, with Ccl8 showing upregulation at both time points and Bpifb1 showing upregulation at acute infection and downregulation at chronic infection. At chronic infection, immunoglobulin components (Ighg, immunoglobulin heavy constant gamma) predominated among upregulated genes, although they were also elevated at acute infection but ranked below chemokines. These gene expression patterns were consistent when comparing acute versus chronic tuberculosis in both tuberculosis-only and co-infected groups (**Supplementary Figure 2**).

For cytokines, this analysis revealed a significant elevation of TNFα and IFNγ levels in tuberculosis-only and co-infected compared to SARS-CoV-2-only lungs at both infection stages, though the adjusted *p*-values for SARS-CoV-2 samples were mostly non-significant in Loupe (**Figure 5**). IL-6 expression was higher in SARS-CoV-2-only lungs, increasing from acute to chronic phases in both SARS-CoV-2-only and co-infected lungs but decreasing in tuberculosis-only lungs in a highly significant manner. IL-4 showed elevated expression in SARS-CoV-2-only lungs at both infection time points, while IL-10 and IL-17a showed increased expression in SARS-CoV-2-only lungs only at the acute time point of infection. IL-13 also showed a significantly increased expression in SARS-CoV-2-only lungs at the acute tuberculosis. This level of induction was not observed during chronic tuberculosis. Finally, IL-22 levels were significantly induced in the tuberculosis-only group, though to a higher level at chronic infection. Taken together, the unique cellular and cytokine profiles associated with each infection or coinfection sample reflected the unique nature of each pathogen with limited similarity in the inflammatory pathways.

### 3.7 Pathway enrichment analysis involved in infection responses

To reveal key pathways activated in different murine lungs from the transcriptome analysis, we conducted pathway enrichment using gene ontology (GO), clusterProfiler, and Gene Set Enrichment Analysis (GSEA) (24, 28–30) on differentially expressed gene sets between infected groups and PBS controls using clusterProfiler’s gseGO package, focusing on immune activation, inflammation, and stress processes. During acute tuberculosis, 67.49% of enriched pathways in co-infected samples were related to immune responses, increasing slightly to 68.50% at chronic infection (**Table 2**). Tuberculosis-only lungs showed a slight decrease of immune-related pathways from acute to chronic stage (~64.35% acute, 61.90% chronic). Interestingly, SARS-CoV-2 samples had a much lower percentage of pathways related to immune response, with 27.50% at acute tuberculosis increasing to 32.68% at chronic tuberculosis. It is important to note that for the SARS-CoV-2 samples, there was high variability in the number of pathways detected during GSEA analysis at both time points of infection. Interestingly, many of the top pathways overlapping between tuberculosis-only and co-infected lung tissues at both acute and chronic time points of infection involved MHC class II complex assembly (GO:0002399), antigen presentation via MHC class II (GO:0019886, GO:0002495), and Fc gamma receptor signaling (GO:0038094). The top 10 pathways for each infection group are shown in **Table 2**. This indicates a bridging between innate immunity and both cellular CD4+ and humoral immunity. However, the number of enriched pathways overlapping between tuberculosis-only and co-infected tissues at chronic infection were much higher at 215 total pathways compared to 59 pathways at acute infection. A subsequent analysis was performed to determine pathways detected in co-infected tissue but not in tuberculosis-only-infected tissue at each infection time point to understand differences in response induced by coinfection with SARS-CoV-2. This analysis showed that the majority of pathways that were unshared between infection types were found in tuberculosis-only-infected tissue (188 pathways at acute tuberculosis and 111 pathways at chronic tuberculosis). These pathways included many T cell processes, including T cell chemotaxis (GO:0010818), positive regulation of T-helper 1 type immune response (GO:0002827), and positive regulation of alpha-beta T cell proliferation (GO:0046641). Conversely, pathways shared between co-infected tissue replicates but not tuberculosis-only-infected tissues were much lower in number (one pathway at acute tuberculosis and 18 pathways at chronic tuberculosis). These pathways encompassed more broadly inflammatory processes and included some cell death signaling. Overall, immune and defense-related pathways were highly activated in co-infected and tuberculosis-only lungs at both acute and chronic infection and were highly similar, while SARS-CoV-2-only-infected lungs showed mainly normal processes such as muscle contraction and cilia movement with some response to viral processes (**Supplementary Figure 4**), suggesting that the overwhelming host responses are geared toward immune and defense responses, most likely to defend against tuberculosis rather than SARS-CoV-2.

**Table 2:** Gene pathway analysis of gene set enrichment analysis (GSEA) using clusterProfiler’s *gseGo*.

Time point post-infection with *M. tuberculosis*	Sample	Go ID	Description	*p* adj.
Acute TB (4 weeks)	TB	GO:0002399	MHC class II protein complex assembly	9.83E-09
		GO:0002503	Peptide antigen assembly with MHC class II protein complex	9.83E-09
		GO:0019886	Antigen processing and presentation of exogenous peptide antigen via MHC class II	9.83E-09
		GO:0002495	Antigen processing and presentation of peptide antigen via MHC class II	9.83E-09
		GO:0002504	Antigen processing and presentation of peptide or polysaccharide antigen via MHC class II	9.83E-09
		GO:0002396	MHC protein complex assembly	9.83E-09
		GO:0002501	Peptide antigen assembly with MHC protein complex	9.83E-09
		GO:0002223	Stimulatory C-type lectin receptor signaling pathway	3.39E-05
		GO:0038094	Fc-gamma receptor signaling pathway	2.91E-08
		GO:0002577	Regulation of antigen processing and presentation	9.83E-09
	SCV2	GO:0001539	Cilium- or flagellum-dependent cell motility	5.93E-08
		GO:0060285	Cilium-dependent cell motility	5.93E-08
		GO:0140374	Antiviral innate immune response	0.0003
	TB/SCV2	GO:0002399	MHC class II protein complex assembly	4.69E-08
		GO:0002503	Peptide antigen assembly with MHC class II protein complex	4.69E-08
		GO:0019886	Antigen processing and presentation of exogenous peptide antigen via MHC class II	1.14E-08
		GO:0002495	Antigen processing and presentation of peptide antigen via MHC class II	1.14E-08
		GO:0002396	MHC protein complex assembly	1.14E-08
		GO:0002501	Peptide antigen assembly with MHC protein complex	1.14E-08
		GO:0038094	Fc-gamma receptor signaling pathway	1.14E-08
		GO:0002504	Antigen processing and presentation of peptide or polysaccharide antigen via MHC class II	1.14E-08
		GO:0002755	MyD88-dependent toll-like receptor signaling pathway	4.80E-07
		GO:0002577	Regulation of antigen processing and presentation	1.66E-06
Chronic TB (16 weeks)	TB	GO:0002399	MHC class II protein complex assembly	4.42E-06
		GO:0002503	Peptide antigen assembly with MHC class II protein complex	4.42E-06
		GO:0019886	Antigen processing and presentation of exogenous peptide antigen via MHC class II	5.83E-09
		GO:0002495	Antigen processing and presentation of peptide antigen via MHC class II	5.83E-09
		GO:0002396	MHC protein complex assembly	5.83E-09
		GO:0002501	Peptide antigen assembly with MHC protein complex	5.83E-09
		GO:0002504	Antigen processing and presentation of peptide or polysaccharide antigen via MHC class II	5.83E-09
		GO:0038094	Fc-gamma receptor signaling pathway	1.20E-07
		GO:0034154	Toll-like receptor 7 signaling pathway	0.0001
		GO:0002478	Antigen processing and presentation of exogenous peptide antigen	5.83E-09
	SCV2	GO:0050764	Regulation of phagocytosis	1.45E-07
		GO:0061041	Regulation of wound healing	9.69E-07
		GO:0002753	Cytoplasmic pattern recognition receptor signaling pathway	3.26E-06
	TB/SCV2	GO:0019886	Antigen processing and presentation of exogenous peptide antigen via MHC class II	1.05E-08
		GO:0002468	Dendritic cell antigen processing and presentation	1.61E-08
		GO:0002495	Antigen processing and presentation of peptide antigen via MHC class II	1.05E-08
		GO:0002399	MHC class II protein complex assembly	0.0001
		GO:0002503	Peptide antigen assembly with MHC class II protein complex	0.0001
		GO:0038094	Fc-gamma receptor signaling pathway	1.05E-08
		GO:0002504	Antigen processing and presentation of peptide or polysaccharide antigen via MHC class II	1.05E-08
		GO:0002579	Positive regulation of antigen processing and presentation	0.0003
		GO:0031665	Negative regulation of lipopolysaccharide-mediated signaling pathway	0.0002
		GO:0002577	Regulation of antigen processing and presentation	1.05E-08

Unfiltered differential expression results comparing treated lung samples to PBS control(s) were used as input for analysis. Total pathways resulting from analysis were filtered to include only those involved in stress and immune response. The top 10 (highest enrichment score) pathways with a set size of 10 or above are shown for each infection group. SARS-CoV-2-only-infected groups had only three overlapping pathways at either time point. Differential expression was performed for each treatment sample compared to the PBS control(s) using Loupe Browser’s differential expression tool (exact negative binomial test). Benjamini–Hochberg (BH) method was used to control false discovery rate, and a *p*-value of <0.001 was used during GSEA analysis. Two lung samples per treatment group were analyzed aside from the acute stage of tuberculosis infection in which three samples were analyzed for the co-infected group and one for the PBS control group.

GSEA, gene set enrichment analysis; PBS, phosphate-buffered saline.

### 3.8 Profile distribution of immune cells in murine lungs

To better reveal the kinetics of changes in localized immunity during infections, we focused our analysis on cell type associated with the dominant immune cell signature of each spot under the tissue of each lung sample at both acute and chronic phases of tuberculosis. Briefly, cell types were annotated using ScType and an ScType murine cell marker database for lung and immune cell types. ScType annotations were verified using Loupe Browser deconvolution gene clusters as well as module scores for each cell type at each cluster. Sccomp was used to determine log ratios of cell type clusters in each treatment compared to the PBS control. A dot plot showing gene markers for each cell type is shown in **Supplementary Figure 3**. At acute tuberculosis, immune cells were broadly distributed throughout the lung in both tuberculosis and co-infected mice (**Figure 6A**). In contrast, during chronic infection, immune cells migrated to deeper lung regions, while SARS-CoV-2-only-infected lungs showed immune cells concentrated near bronchial entrances (**Figure 6B**). Additionally, B cells localized in tight clusters of inflammation at chronic tuberculosis infection, potentially forming tertiary lymphoid structures, especially in tuberculosis-only-infected tissues. The inflammatory infiltrates in tuberculosis and co-infected lungs encompassed diverse immune cell populations, including B cells, natural killer (NK) cells, activated and inflammatory macrophages and monocytes, interstitial macrophages, and myeloid cells (Dataset S3, S4). Conversely, SARS-CoV-2-only-infection was characterized primarily by IFN response cells during acute infection, shifting to macrophages and NK cells at the chronic tuberculosis. Non-immune cell populations were more consistent across groups and time points, whereas immune cell abundances varied substantially between tuberculosis-infected groups and SARS-CoV-2-infected groups (**Figure 6C**). Macrophages, monocytes, NK cells, IFN response cells, and myeloid cells were considered innate immune cell types, while B cells were considered adaptive immune cell types. All other detected cell types were categorized as non-immune/healthy cells.

Functional-cell-type analysis revealed that a larger proportion of lung tissue in SARS-CoV-2 samples consisted of non-immune cell types, such as alveolar type II cells, compared to tuberculosis-only samples (**Figure 6C**). Tuberculosis-only-infected tissues had 8.2% more healthy cell types at acute tuberculosis but had 17.4% more inflamed cell types at chronic tuberculosis. In co-infected lungs, healthy cells represented only 1.0% more than inflamed cells at acute infection, while inflamed cells made up 23.4% more than healthy cell types at chronic infection. Immune cell populations were relatively sparse in SARS-CoV-2-only-infected lungs compared to tuberculosis-infected groups at both time points. It is noteworthy to mention that all infected lungs exhibited a dominant innate immune response over adaptive immunity at both times, primarily showing pronounced signatures of macrophages especially in tuberculosis-infected tissues (**Figure 6C**). Differential composition analysis revealed that inflammatory and activated macrophages as well as B cells were enriched in both tuberculosis only and co-infected lungs at acute infection, especially in tuberculosis-only-infected tissue, whereas these cell types were depleted in SARS-CoV-2-only-infected lungs. In contrast, SARS-CoV-2-infected lung tissues showed an enriched IFN response cell population at acute infection which was not seen in tuberculosis-infected lungs (**Figure 7A**). A similar pattern was seen at chronic tuberculosis with enriched populations of macrophages in both co-infected and tuberculosis-infected tissues and enriched B cell populations seen especially in tuberculosis-only-infected lungs (**Figure 7B**). T cell populations were not dominant at either time point of infection, with B cells dominating the adaptive response. Though T cell signatures were not dominant enough to form a distinct cluster at either time point, module scoring was used to detect which annotated cell type clusters had the highest CD4 and CD8 T cell signal. Overall, B cell clusters in both tuberculosis-only- and co-infected tissues at chronic infection had relatively higher module scores (~0.26) for CD4 signal than other clusters, indicating a potential for co-localization of CD4 T cells with the dominant B cell signature in these clusters. Clusters of inflammatory and activated macrophages also had elevated module scores for both CD4 and CD8 T cells at both time points of infection (~0.1 to 0.29) compared to other non-immune clusters, also indicating a potential for co-localization of T cell subsets with macrophages in areas of inflammation. In conjunction with module scores, CD4 T cell signals in macrophage clusters had high percent positive values in tuberculosis-only-infected tissues (82.5% to 94.9%), indicating that a high percentage of macrophage clusters also carried a positive CD4 T cell signature.

### 3.9 Moran’s I spatial autocorrelation and macrophage phenotype during superinfection

**Figure 8:**
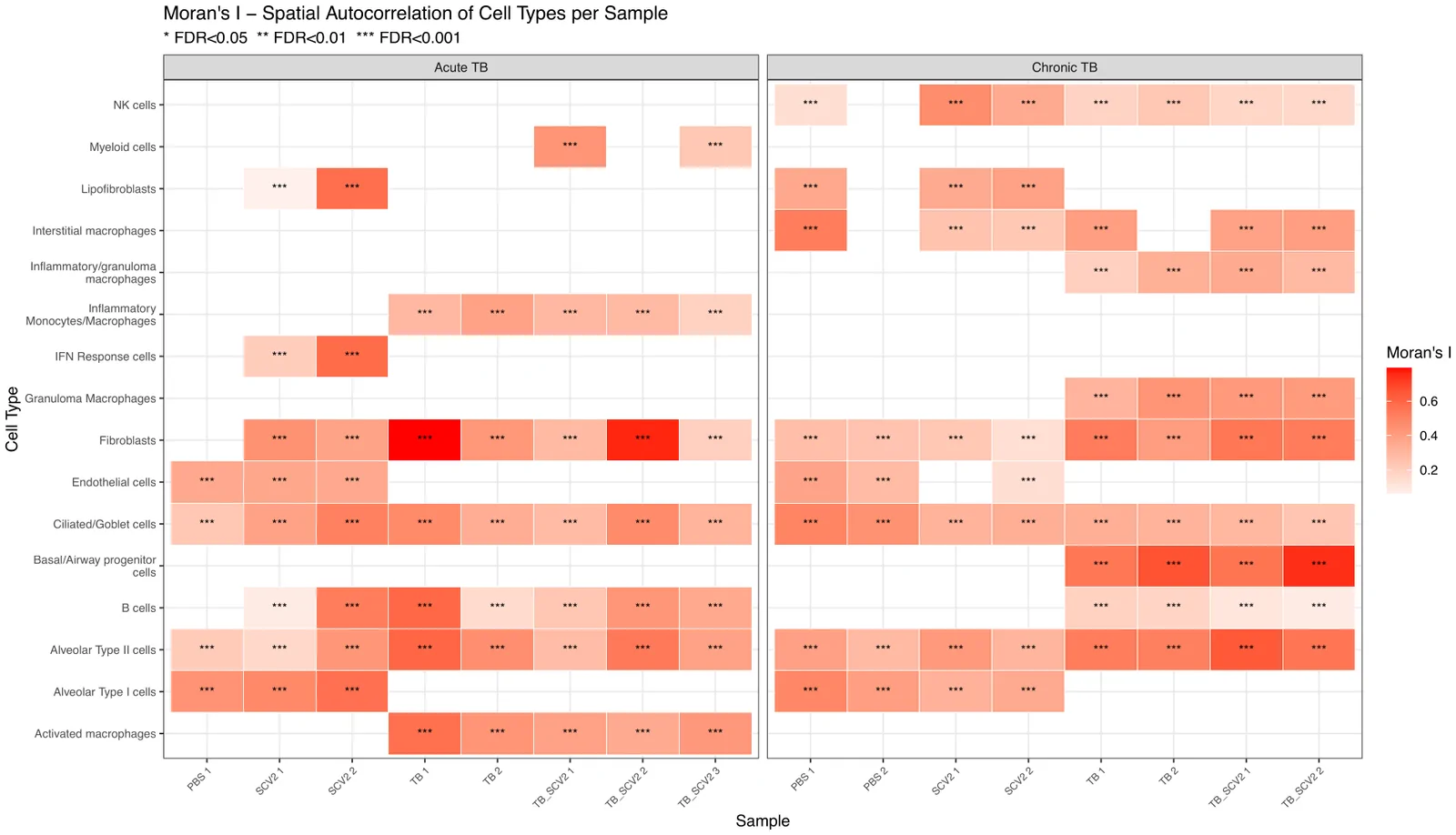
Moran’s I spatial autocorrelation. Moran’s I was calculated using R for each treatment at each time point of infection with *M. tuberculosis*, acute and chronic. A radius multiplier of 1.5 was used. Spatial correlation of cell types was considered significant if FDR<0.05 (*), FDR<0.01 (**), or FDR<0.001 (***). Moran’s I uses spatial autocorrelation to produce an index value between -1.0 (perfect spatial dispersion) and +1.0 (perfect spatial clustering). FDR = false discovery rate.

To further explore how well different cell types clustered spatially, Moran’s I was calculated for each sample and cell type. Overall, the major expression profile of these lesions showed dominantly B cells especially in tuberculosis-only-infected lungs at chronic infection (**Figure 6B**), a major player in host adaptive immune responses to tuberculosis (31, 32). However, Moran’s I calculations showed that spatial clustering of B cells was highly variable, with some tuberculosis and co-infected lungs having higher Moran’s I values than others, indicating varied response (**Figure 8**). Interestingly, B cell Moran’s I values ranged from about 0.15 to 0.59 (FDR = 7.84E-107 to 0) at acute tuberculosis and 0.11 to 0.19 (FDR = 3.56E-132 to 0) at chronic tuberculosis, indicating more spatial correlation of B cells at acute infection. The most highly spatially correlated cell types made up mostly non-immune cell types, including fibroblasts at acute infection (Moran’s I 0.76 to 0.79, FDR = 0) and basal cells at chronic infection (Moran’s I 0.66 to 0.74, FDR = 0). Though most Moran’s I values were low, indicating less definitive spatial clustering of cell types. They were positive for all cell types in a given sample, which is expected for biological tissues.

**Figure 9:**
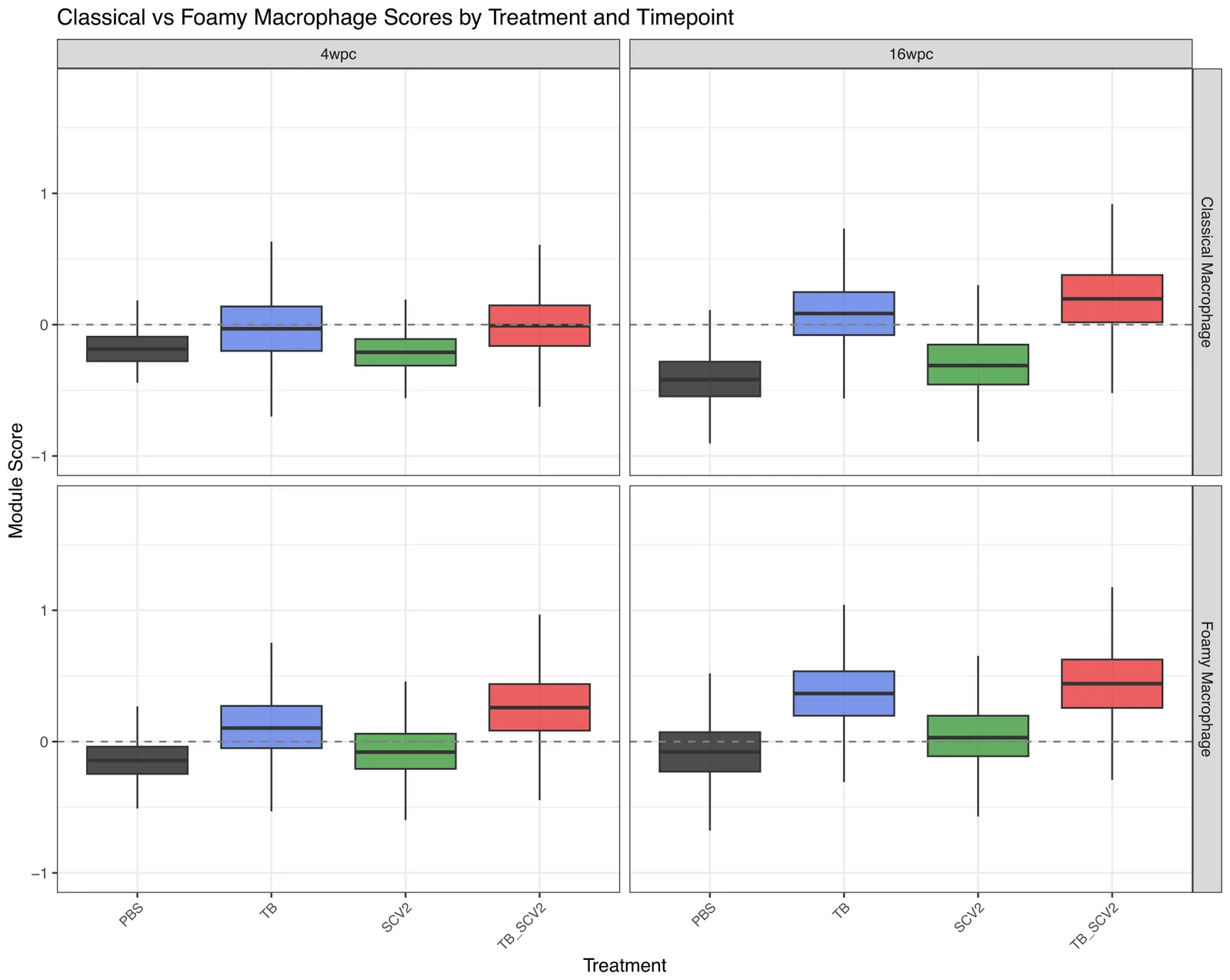
Macrophage phenotypes represented in murine lungs with different infections. Module scores were calculated using R (Add Module Score, average expression subtraction) with classical macrophage markers: Adgre1, Cd68, Cd11b (Itgam), Mrc1, Cd14 and Csf1r, or foamy macrophage markers: Apoe, Plin2, Plin3, Lpl, Fabp4, Trem2 and Lipa. A set of random control genes (n=50) were used to subtract background. Higher module scores indicate the treatment group expresses that macrophage subset more as compared to the background gene set. Two lung samples per treatment group were analyzed, aside from the acute stage of tuberculosis infection in which 3 samples were analyzed for the co-infected group and 1 for the PBS control group. PBS = phosphate buffered saline

**Figure 10:**
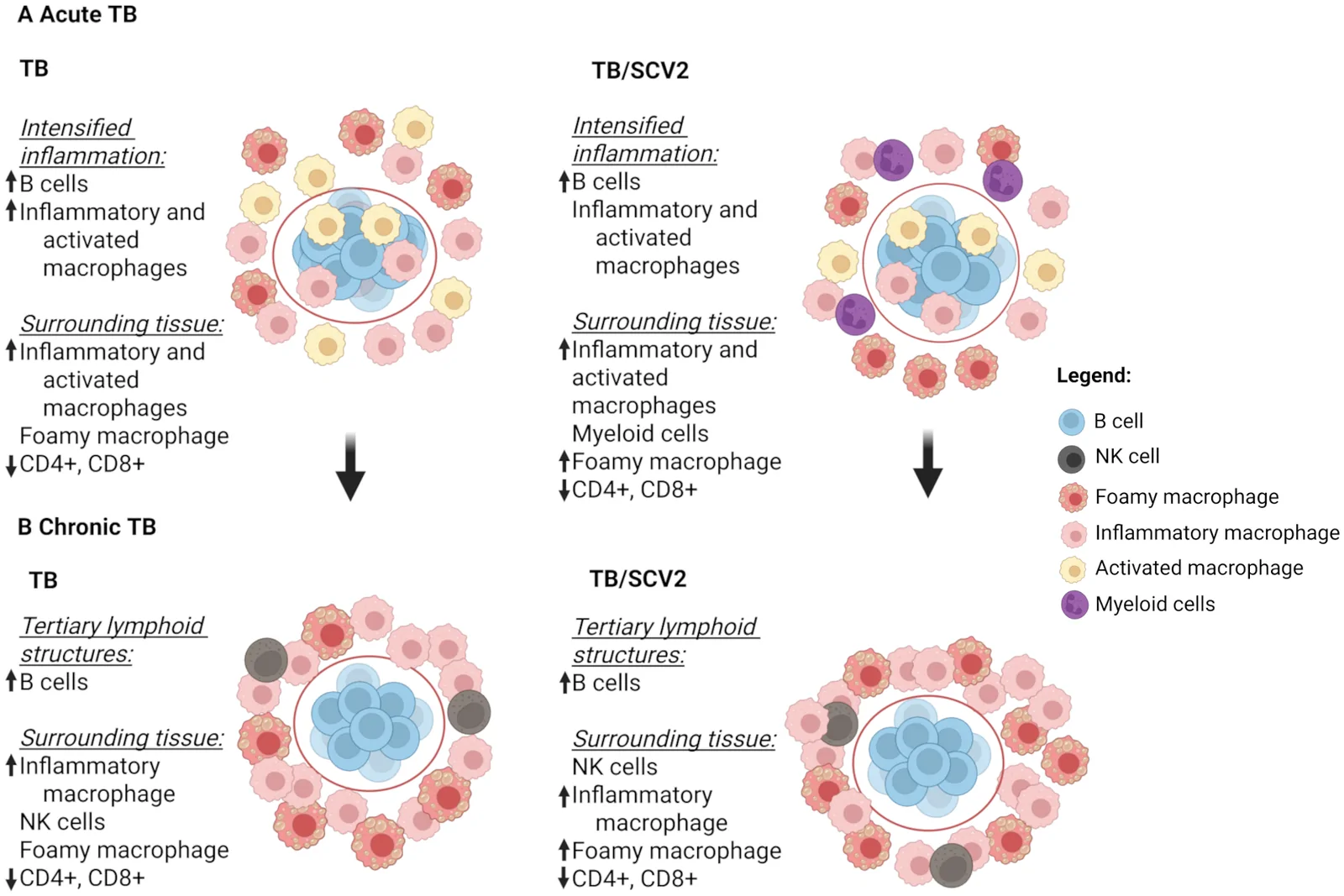
Summary sketch depicting overall analysis of cell types identified during spatial RNA sequencing of murine lungs superinfected with SARS-CoV-2 at both acute and chronic tuberculosis. Cell types were divided into areas of intensified inflammation or tertiary lymphoidstructures and surrounding tissue. Intensified inflammation/tertiary lymphoid structures are circled in red in the figure. Each figure shows an average composite of replicates from each treatment (TB or TB/SARS-CoV-2). Cell types shown with an arrow indicate cell types that were more highly/less represented in that tissue treatment. Figure made using BiorenderTM. Two lung samples per treatment group were analyzed, aside from the acute stage of tuberculosis infection in which 3 samples were analyzed for the co-infected group.

Finally, a more targeted analysis of macrophage populations within lung sections to examine spots expressing marker gene sets corresponding to classical macrophages and foamy macrophages revealed distinct differences in these populations between infection time points (**Figure 9**). Overall, the module scores of classical and foamy macrophages increased from acute to chronic tuberculosis in tuberculosis-infected and co-infected lung tissues. The module scores of foamy macrophages were highest in co-infected tissues, especially at chronic infection, indicating a shift toward a lipid-laden macrophage phenotype. The module scores of both macrophage populations remained very low or negative for SARS-CoV-2-infected and PBS lungs, suggesting that macrophage recruitment is a host response mainly to *M. tuberculosis* infection. Overall, cellular profiling results from clustering, macrophage population, and Moran’s I analysis were combined to summarize the overall findings from spatial RNA sequencing analysis of murine lungs from the different examined groups (**Figure 10**).

## 4 Discussion

The clinical data of *M. tuberculosis*/SARS-CoV-2 infections show that patients with a prior *M. tuberculosis* infection will have worse outcomes in almost all facets of recovery and outcome, including shorter times to death, longer times to recovery, and greater mortality (4, 5, 33–35). In a recent cohort study, a dual tuberculosis/COVID-19 diagnosis resulted in 10% higher mortality than in patients only infected with COVID-19 (36). However, in murine infection studies published on tuberculosis/SARS-CoV-2 coinfections, prior *M. tuberculosis* infection was protective against high SARS-CoV-2 levels in the lung and does not result in higher mortality (15, 16). In this study, we sought to determine if tuberculosis disease severity, modeled using a higher dose of *M. tuberculosis* with a virulent strain (Erdman), would change the mortality outcomes at early (acute) and late (chronic) stages of tuberculosis. In our hands, the higher dose of *M. tuberculosis* resulted in 100% mortality of the co-infected group, a much higher rate than that observed in the human cohort study (36), but only during acute tuberculosis. Interestingly, this difference was not seen at the later, chronic stage of tuberculosis, nor in a prior study that examined the effect of a low dose of *M. tuberculosis* (16). Earlier coinfection studies demonstrate that prior *M. tuberculosis* infection limits SARS-CoV-2 replication because of the preexisting inflammatory response when the virus arrives (15–17). Similarly, the acute *M. tuberculosis* infection with a high dose reported here resulted in no detectable viral loads, lower than those seen in other studies with a more moderate dose of ~100 CFU/lung (16, 17). However, this did not result in reduction of mice mortality rate. Viral loads increased instead in the brain relative to the control SARS-CoV-2 group and resulted in 100% mortality during acute tuberculosis. This is, in part, a consequence of the hACE2-transgenic mouse model which over-expresses hACE2 in the brain, leading to an acute infection resulting in encephalitis and neuronal damage seen rarely in clinical human infections (19, 37). All mice experiencing mortality had viral loads in the lung of ≥6 logs. Interestingly, the lung and brain protection hinged on systemic neutralizing antibody responses over local SARS-CoV-2-specific cytokine production in prior studies (27, 38). From these observations, we concluded that the increased mortality at acute tuberculosis could be mediated by the high level of SARS-CoV-2 in the brains of K18 hACE2 mice as indicated before (19) and not by the high dose of *M. tuberculosis* used in this experiment. Nonetheless, when mice were superinfected with SARS-CoV-2 at chronic tuberculosis, the viral loads in brains of infected animals were, on average, two logs lower than those observed in acute tuberculosis and hence were not associated with mortality, most likely due to the heightened immune responses associated with chronic tuberculosis as indicated before (39–42) and as shown by RNA sequence analysis later on. This phase-dependent divergence in viral loads may reflect the differing mortality patterns observed between the co-infected groups at acute versus chronic stages of tuberculosis. To our knowledge, this is the first study demonstrating that a potential respiratory tract immune environment drives SARS-CoV-2 to the brain, resulting in fatality only during acute coinfection with another respiratory pathogen.

While the mouse model used partially confounds the presented results, neuro-clinical manifestations of SARS-CoV-2 have been recorded in humans. In fact, up to 80% of hospitalized patients had some neurological symptoms at some point in their disease progression, with severe COVID-19 disease being a main predictor (43–45). More concerning is the fact that there have been two clinical case studies of tuberculosis/SARS-CoV-2 resulting in central nervous system tuberculosis (46, 47). One of these studies is in an adult, where central nervous system tuberculosis incidence is less than 1% of total cases (48). In this study, we were surprised to isolate bacterial colonies from the brain in both the tuberculosis/SARS-CoV-2 and tuberculosis-alone groups. The tuberculosis/SARS-CoV-2 groups had greater mycobacterial load in more animals, although not statistically different from the control tuberculosis-alone group (**Figure 2**). An earlier study using a high-aerosol-dose model of tuberculosis also showed detectable central nervous system (CNS) invasion, albeit in a different mouse model with a different strain of tuberculosis (49) than the Erdman strain used here.

Interestingly, histopathological analysis revealed more inflammatory responses in chronic tuberculosis compared to acute tuberculosis but with a demonstrated less organized cellular composition throughout the superinfected lungs compared to tuberculosis-only-infected mice at both acute and chronic time points. This observation indicates that SARS-CoV-2 can modulate tuberculosis lung pathology, with consequences including disruption of organized inflammatory responses, as suggested before (50–52). Given that SARS-CoV-2 did not reach the lungs at a detectable level during acute tuberculosis, the greater changes observed between groups at the chronic phase may be due to actual SARS-CoV-2 invasion. Overall, histology of murine lungs indicated differential host responses that uncovered several questions related to the nature and type of cells constituting those types of reactions. Additionally, there were no statistical differences in any tissue at either time point in this study for bacterial burden. Bacterial burden data may be partially obscured due to the high tissue loads already in existence, which was consistent when others used *M. tuberculosis* H37Rv strain for superinfection (15, 16). Additionally, the high infectious dose administered could, in part, be the cause for the extreme immune dysregulation seen in all tuberculosis-infected tissues.

To further characterize host responses in murine lungs, the target organ for both tuberculosis and SARS-CoV-2, we employed an approach dependent on RNA next-generation sequencing (RNA-seq) employing Visium CytAssist (10x Genomics) to further dissect the host transcriptomic differences and cellular defenses among groups in a spatial context. The results from spatial sequencing in this study indicated that, overall, inflammation-regulated genes were, on average, equal between tuberculosis-only and co-infected tissues during acute tuberculosis. UMAP plots showed a trend of more similar cell type clustering between PBS and SARS-CoV-2 samples and between tuberculosis-only and co-infected tissues at chronic infection, indicating that a balance was reached at chronic infection, where tuberculosis was the driving factor in the inflammation status of the lung. At both infection time points, total inflammation was higher in superinfected compared to tuberculosis-only groups. In addition, cytokine profiles indicate an overall pro-inflammatory response in both superinfected and tuberculosis-only-infected tissues, with an increased expression of key genes representing pro-inflammatory cytokines such as TNFα and IFNγ. Both TNFα and IFNγ are considered essential in controlling *M. tuberculosis* disease progression (53). Both cytokines were elevated in the superinfected and tuberculosis-only-infected lungs and significantly lower in the SARS-CoV-2-only-infected tissues at both acute and chronic tuberculosis. In an earlier report using a different mouse/SARS-CoV-2 model system (strain 129S1 wild-type mice infected with the severe B.1.351), the IFNγ levels were increased after reinfection (54), unlike the K18-jACE2 strain used in this study, which is another indication of the impact of host genetics on infection outcome. The levels of IL-22 were higher in the tuberculosis-only-infected lung samples, lower in both superinfected and SARS-CoV-2-only-infected samples at acute infection, and even more marked at chronic infection. Since IL-22 has been shown to provide protection against tissue damage that can be conferred by pro-inflammatory cytokines like TNFα (55), the increase in the expression of IL-22 may have helped in maintaining survival in tuberculosis-only-infected mice at chronic infection, but not in other groups, including superinfection at both acute and chronic tuberculosis.

Gene expression analysis indicated that more genes overlapped between tuberculosis only and co-infected lungs at both time points of infection. This pattern was also seen when comparing between time points, indicating that tuberculosis remains dominant in immune response regulation. Interestingly, no genes overlapped between SARS-CoV-2 samples and either tuberculosis-only or co-infected samples when comparing between time points. Several genes were upregulated between all treatment groups at acute infection, with Ccl8 and Bpifb1 showing differing expressions at chronic infection as well. Ccl8 has been shown to be highly upregulated in tuberculosis cases, acting as a recruiting agent for innate immune cells (56). It has also been considered as a potential marker for differentiation between acute and latent stages of tuberculosis. Indeed the Ccl8 expression levels were, on average, around 4 logs higher at acute infection than at chronic infection for both tuberculosis only and co-infected groups. Though the role of Bpifb1 has been studied in cystic fibrosis, chronic obstructive pulmonary disease, and various carcinomas (57, 58), it has not been widely studied in tuberculosis infection. Conversely, at chronic infection, Bpifb1 was downregulated, indicating a shift away from its utilization over time of infection. Bpifa1 was the only gene overlapping between SARS-CoV-2 and co-infected samples at acute infection and was upregulated. Bpifa1 is involved in acute immune response and neutrophil infiltration and has been found to be upregulated during moderate and severe cases of SARS-CoV-2, albeit in human cases (59). Finally, Myh6, Ccl7, Irf7, and Ccl12 were the only genes overlapping between SARS-CoV-2 and tuberculosis-only-infected tissues at acute infection and were all upregulated. These genes could also all have roles in immune response.

At chronic tuberculosis, the genes overlapping between all infections were unrelated to immune response and downregulated, namely: Kap, Slc34a1, Acsm2, Miox, Slc22a8, and Akr1c21. Folr1, a folate receptor, was the only gene overlapping between SARS-CoV-2 and co-infected samples and was downregulated. Folate has been correlated with efficient viral replication, and thus targeting or, in this case, downregulation of Folr1-mediated intake of folate into the cell could be important in reducing SARS-CoV-2 proliferation as reported before (by Gowda and Sen, preprint (60)). Gzmk was the only gene overlapping between SARS-CoV-2 and tuberculosis-only-infected tissues at chronic infection, though it was upregulated in tuberculosis-infected tissues and downregulated in SARS-CoV-2-infected tissues. It was also upregulated in tuberculosis-infected tissues at acute infection. Gzmk is a granzyme produced by cytotoxic immune cells, including NK cells and some T cells in response to inflammation (61), making it important in immune response against tuberculosis. A study on early infection with SARS-CoV-2 in humans also found that Gzmk was downregulated (62), similar to the profile identified here.

Pathway enrichment analysis performed using GSEA indicated a high proportion of Gene Ontology (GO) pathways related to immune response at both acute and chronic time points of infection for tuberculosis only and co-infected tissues and a lower percentage of pathways related to immune response for SARS-CoV-2-only-infected tissues at both time points. This result was consistent with overall higher inflammation in tuberculosis only and co-infected lung tissue at both infection time points, though this could be conflated, in part, by the high dose of *M. tuberculosis* administered. On average, the percentage of pathways involved in immune response increased slightly in co-infected samples from acute to chronic stages of infection but decreased slightly in tuberculosis-only-infected samples from acute to chronic infection. However, overall, the percentages of pathways involved in immune function remained similar between tuberculosis-only and co-infected tissues across both time points of infection. Fewer GO pathways involved in biological process were detected in SARS-CoV-2-infected samples at both time points of infection, indicating that, overall, infection with tuberculosis was more significant in producing higher numbers of enriched pathways. This result was consistent with the volcano plots shown in **Supplementary Figure 2** that indicated fewer differentially expressed genes in SARS-CoV-2 samples at both time points of infection. Additionally, comparisons between pathways detected in co-infected tissues but not in tuberculosis-only-infected tissues resulted in very few pathways, supporting that tuberculosis seems to be the major contributor to pathways of inflammatory process whereas SARS-CoV-2 has a lesser effect.

In another level of analysis, we used gene transcripts as markers for cellular composition of murine inflammatory responses among infected groups. Surprisingly, this analysis identified a relatively high percentage of B cells present in lung tissues at acute infection, and though lower numbers of B cells were seen at chronic infection, they were spatially organized over areas of intense inflammation, especially in tuberculosis-only-infected lungs. Though B cells were compositionally enriched in these clusters and had positive Moran’s I values, the Moran’s I values ranged from 0.15 to 0.59 for tuberculosis-infected tissues at acute infection and 0.11 to 0.19 at chronic infection, indicating that B cells may have been distributed across the tissue as well as clustering. Notably, populations of T cells were not identified as dominant cell types at either time point of infection. T cells have been shown to play a vital role in the control of tuberculosis and are important in the formation of granuloma structures to prevent the dissemination of the pathogen (63). However, more recent studies have shown that B cells and plasma cells may confer protection against tuberculosis and have been shown to form germinal centers (GC) within the follicular area of the granuloma (32, 64, 65). B cells can also aggregate into tertiary lymphoid structures in the lung following infection with *M. tuberculosis*, potentially playing a protective role in later stages of the disease (66). Indeed B cells were rich in areas of necrosis in both tuberculosis and superinfected lungs, especially at the chronic stage of infection, indicating that ectopic GC or more mature tertiary lymphoid structures may have been forming in those areas to combat the infection. The B cells in GC are important for regulating the immune response to tuberculosis by producing antibodies and other cytokines, especially during acute infection. At chronic infection, B cells may help with reducing extreme inflammation and fibrosis. Additionally, B cells may help promote the formation of granulomas (64). Overall, research is limited on the role of B cells in tuberculosis infection. This study indicated pronounced expression profiles for B cells in areas of intense inflammation, especially at chronic infection. Further research in other models that are more susceptible to tuberculosis could assist in the understanding of the roles of B cells and humoral immunity in tuberculosis infection and SARS-CoV-2 coinfection along with the cell-mediated immune response. Additionally, incorporating anti-CD20 depletion, B-cell-deficient mice, neutralizing antibody measurement by ELISA, and IFN-γ ELISpot methods would be necessary to confirm whether B cells played a protective role. These methods could be employed in future coinfection.

Interestingly, T cell populations were not dominant enough to form their own cell type cluster at either time point of infection. However, module scoring of T cell markers indicated that T cell signals co-localized with many of the dominant macrophage and B cell cluster populations. T follicular helper cells, a subset of CD4 T cells, are important in the development of B cells and production of antibodies and can be found in tertiary lymphoid structures with B cells (67). Indeed the module score for CD4 T cell signal was elevated in B cell clusters of tuberculosis-only and co-infected lungs mainly at chronic infection, indicating the potential for more mature tertiary lymphoid structure formation in tuberculosis-infected lung tissue at the later time. Histological confirmation using markers such as CD21/CD23 as well as immunofluorescence and spatial proteomic validation methods would be needed to confirm the presence of tertiary lymphoid structures. T cells are also essential in detecting macrophages infected with *M. tuberculosis* (68). Both CD4 and CD8 T cell module scores were increased in tuberculosis-only and co-infected tissue at both times of infection, indicating tight co-clustering and interplay between macrophage and T cell populations even in the more dominant macrophage-annotated clusters. Though module scoring showed potential for the co-localization of T cell subsets with other cell types, this does not confirm distinct T cell clusters.

Beyond B cell and T cell populations, the presence of both classical and foamy macrophages was also analyzed. Macrophages are essential in phagocytosis and processing of intracellular pathogens like *M. tuberculosis* and formation of granulomas (69). Although module score gene signatures of both foamy and classical macrophages were increased in tuberculosis and co-infected lung tissues at both acute and chronic infection, their locations were dispersed across the tissue rather than localized in areas of intense inflammation. This result aligned with the widespread macrophage populations seen in both tuberculosis-infected groups. Foamy macrophages are thought to be a factor in chronic inflammatory responses caused by *M. tuberculosis*, which could be why populations of foamy macrophages were increased from acute to chronic infection in both tuberculosis- and co-infected lung tissues (70). This chronic inflammation could then contribute to the recruitment of classical macrophages to help control the infection. Indeed classical macrophage populations were also increased from acute to chronic infection in tuberculosis-only and co-infected tissues. During chronic tuberculosis, both classical and foamy macrophages were especially present in the SARS-CoV-2 co-infected group, suggesting enhanced recruitment of active macrophages to balance the lipid-laden foamy macrophage populations in hosts suffering from chronic tuberculosis.

In summary, a high dose *M. tuberculosis* Erdman infection had variable impacts on the host when concomitant with a SARS-CoV-2 infection dependent on the phase of tuberculosis infection. On both transcriptomic and cellular levels, the presented analysis provides a view of dynamic changes associated with transition from acute to chronic tuberculosis, especially when a host is superinfected with SARS-CoV-2. This comprehensive analysis of cellular dynamics reveals that superinfection with SARS-CoV-2 could regulate the immune response during chronic tuberculosis, particularly affecting B and T cell populations. More direct measurement of CD4 T cell subsets would require cytokine staining methods, which could be employed in subsequent studies. Future studies could utilize spatial RNA sequencing in other models such as C3HeB/FeJ mice, where human-like granulomas could be analyzed during superinfection. Additionally, the use of other SARS-CoV-2 variants such as Omicron could benefit future studies by lending a different coinfection phenotype. Expanding the duration of infection past 16 weeks could provide additional insight into whether the immune changes seen here are reversible over longer times of recovery. Moreover, increasing the number of mouse lung samples analyzed by spatial sequencing would lead to more robust results. In addition, comparing spatial sequencing to other approaches that target host cellular responses (such as flow cytometry, immunofluorescence, or single-cell RNA sequencing) could complement this emerging technology. Furthermore, the use of a lower dose of *M. tuberculosis* (50–100 CFU/lung) in future studies could more accurately represent natural exposure and help separate dose-dependent from coinfection-specific changes in immunity. Finally, the insights gained from the exploratory cellular and genetic analyses provided in this study could help in revealing novel mechanistic pathways active during tuberculosis infection and coinfection with SARS-CoV-2 or any other viral respiratory pathogens. Those insights could be further exploited to develop effective vaccines and chemotherapies to help patients grappling with both infections.

## Data Availability

The datasets have been submitted for release and should now be available for searching under BioProject: PRJNA1374175.

## Supplementary material

Supplementary Figure 1 QC metrics from SpaceRanger aggregate analysis of samples showing gene and UMI counts per spot. The y-axis is in log scale and represents counts +1. UMI, unique molecular identifier.

Supplementary Figure 2 Spatial RNA-seq gene expression analysis. Volcano plots showing overlaid upregulated, downregulated, and non-significant genes for each treatment at acute and chronic tuberculosis. Differential expression was performed for each treatment sample compared to the PBS control(s) using Loupe Browser’s differential expression tool (exact negative binomial test). Differential expression gene sets were filtered to include only genes with log2 fold change of >2.0 or <-2.0 and a p-value of <0.001. PBS, phosphate-buffered saline.

Supplementary Figure 3 Spatial RNA-seq cell type analysis. Dot plot depicting marker genes corresponding to cell type. Made using Seurat DotPlot function with assay “SCT” to scale and plot Pearson residuals.

Supplementary Figure 4 Spatial RNA-seq pathway enrichment analysis comparing the differential expression of treatment groups between acute and chronic time points of tuberculosis infection. **(A)** TB only. **(B)** SARS-CoV-2 only. **(C)** TB/SARS-CoV-2. Analysis completed using clusterProfiler’s gseGO package where gene ratio is calculated as the total count of genes in a differentially expressed gene set divided by the total count of genes in a pathway gene set, and a p-value of 0.001 was selected. Differential expression was performed for each treatment sample compared to the PBS control(s) using Loupe Browser’s differential expression tool (exact negative binomial test). PBS, phosphate-buffered saline.
